# Synthesis of copper nano/microparticles via thermal decomposition and their conversion to copper oxide film

**DOI:** 10.55730/1300-0527.3565

**Published:** 2023-05-09

**Authors:** Çağdaş ALLAHVERDİ

**Affiliations:** Department of Software Engineering, Faculty of Engineering, Toros University, Mersin, Turkey

**Keywords:** Thermal decomposition, copper, copper oxide, nanoparticle, Feret’s diameter, Tauc’s method

## Abstract

Copper nano/microparticles were synthesized in octadecene at 290 °C by thermal decomposition method. Copper acetate monohydrate, stearic acid, and 1-octadecanol were used as copper precursor, capping and mild reducing agents, respectively, at the synthesis. Borosilicate glass substrates submerged into the reaction solution during the synthesis were coated by copper nano/microparticles. Thermal decomposition and coating techniques were combined in this study. Copper nano/microparticles were thoroughly characterised via X-ray powder diffraction, X-ray photoelectron, Raman, and attenuated total reflectance-Fourier transform infrared spectroscopies, and scanning and transmission electron microscopies. The average minimum Feret’s diameter of these synthesized copper particles was measured as ~87 ± 19 nm. Copper nano/microparticles were converted to copper oxide nano/microparticles by applying heat treatment at 250 °C. The phase composition of copper oxide nano/microparticles was determined by reference intensity ratio analysis. The energy gap of copper oxide nano/microparticles was determined as ~2.33 eV by using Tauc’s method. Their band gap PL emission was observed at ~2.15 eV.

## 1. Introduction

Group IB metals (copper, silver, and gold) are very important in our daily life. We are using these precious metals in a variety of fields such as jewellery, electricity and electronics, and medicine, etc. Copper’s advantage comes from its abundance in the earth’s crust compared with silver and gold, thereby it is cheaper and mainly used for wiring. Nanotechnology applications of copper nano/microparticles such as the reduction of carbon dioxide [1], superhydrophobic surfaces [2], and conductive patterns [3,4] have attracted much attention. Copper is known as an antimicrobial material for centuries. Today, we know that many types of bacteria, for instance, Salmonella enterica, Escherichia coli, and Staphylococcus aureus, can be killed in a few minutes or hours when their capsule or cell wall is in contact with a copper surface [5]. Stainless steel push plates were coated with copper microparticles (between 5–60 μm) by Hutasoit et al. [6]. They reported 99.2% SARS-CoV-2 virus reduction at 5 h on the copper-coated push plates. Many researchers indicate that copper is sensitive to oxidation more than silver and gold due to its lower reduction potential [7,8]. Effenberger et al. synthesized copper nanoparticles by decomposing copper acetate in diphenyl ether with oleylamine, oleic acid, and 1,2-octanediol above 220 °C [9]. They showed that the oleic acid ligands coating the copper nanoparticles reduced the antibacterial effect compared to the bare ones but protected the copper nanoparticles against air oxidation.

Park et al. published a thermal decomposition procedure for synthesizing monodisperse nanocrystals [10]. According to their procedure, metal chloride and sodium oleate can be reacted to form a metal-oleate complex and thus this complex can be thermally decomposed into monodisperse nanocrystals in a high boiling point solvent. They synthesized 40 g of monodisperse iron oxide (magnetite) nanocrystals by heating iron-oleate complex slowly in 1-octadecene up to 320 °C. Various copper complexes such as copper-oleate [11–13], copper cupferrate [14], copper oxalate [15,16], copper acetylacetonate [17,18], bis(salicylaldiminato)copper(II) [19], N,N-diethyl-diaminopropane-copper(II) oxalate [20], copper-bis(1(2)H-tetrazol-5-yl)amine [21], bis(triphenylphosphine)copper(I) 2-(2-(2-methoxyethoxy)ethoxy)acetate [22], and copper(II) dialkylamino alkoxide [23] were thermally decomposed to produce copper nanoparticles in solvent or solvent-free media.

In this study, copper nano/microparticles have been successfully synthesized in 1-octadecene. To produce pure copper nano/microparticles, formation of copper stearate intermediate and its thermal decomposition at 290 °C have been performed in 1-octadecene under pure argon. 1-octadecanol has been used as both organic dispersant and mild reducing agent at the synthesis. Copper nano/microparticles have been accumulated on borosilicate glass discs submerged in 1-octadecene during the synthesis. Thus, for the first time, both syntheses of copper nano/microparticles and their coating on glass have been achieved in one-pot without needing to use time-consuming processes or expensive and complex systems. Nonfused pure copper nano/microparticles have been observed under a magnification of 100,000×. Copper nano/microparticles have been converted to copper oxide nano/microparticles by the heat treatment process. The crystalline phase, forbidden energy gap, and band gap luminescence of these copper oxide nano/microparticles have been investigated by means of X-ray powder diffraction, optical transmission, and photoluminescence spectroscopies, respectively.

## 2. Experimental

### 2.1. Materials

Copper(II) acetate monohydrate (Cu(CO_2_CH_3_)_2_·H_2_O, 99.99%), stearic acid (CH_3_(CH_2_)_16_COOH, 95%), 1-octadecanol (CH_3_(CH_2_)_17_OH, 99%), 1-octadecene (CH_3_(CH_2_)_15_CH=CH_2_, 90%), methanol (CH_3_OH, ≥99.9%), toluene (C_6_H_5_CH_3_, ≥99.9%), copper powder (Cu, 99.5%, <425 Mm), copper(II) oxide powder (CuO, 98%, <10 Mm) and copper(I) oxide powder (Cu_2_O, ≥99.99%) were bought from Merck. D 263 M circular cover glasses with a diameter of 10 mm were used in the experiment. They are flat, colourless, and chemically resistant borosilicate glasses. Light transmission of these D 263 M glasses is greater than 90% between 350–800 nm for a thickness of 0.15 mm.

### 2.2. Methods

**Figure 1:**
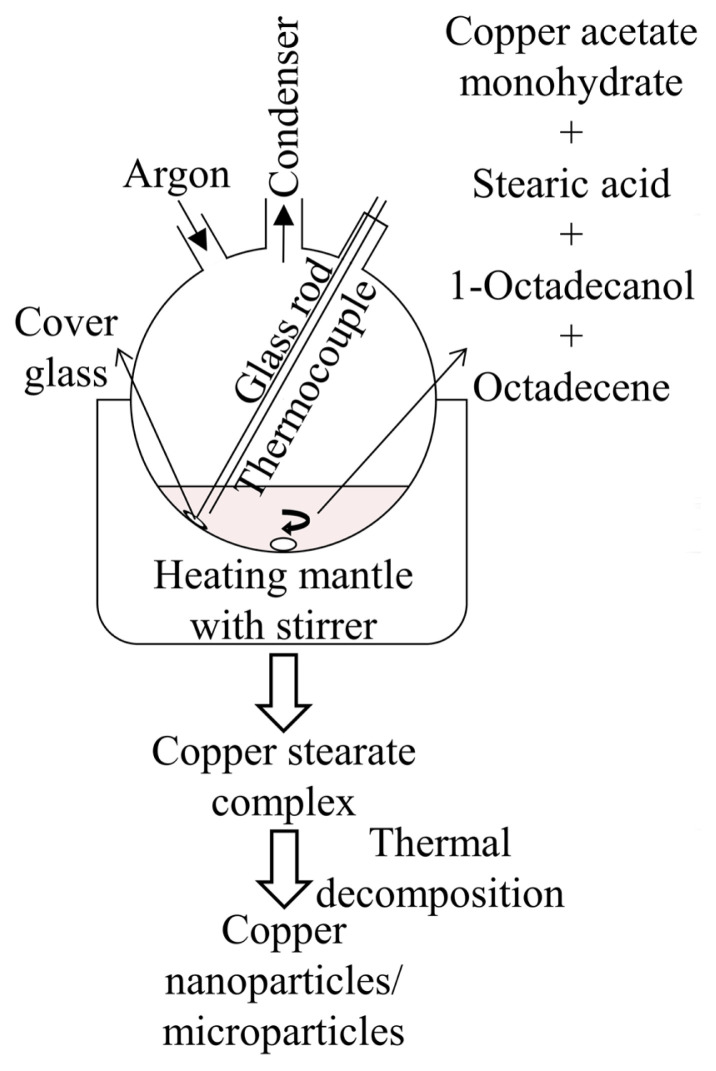
Synthesis of copper nano/microparticles by means of thermal decomposition. Copper nano/microparticles are produced and deposited on cover glass submerged in octadecene.

A schematic representation of the formation of copper nano/microparticles is shown in Figure 1. Thermal decomposition method was utilized to synthesize copper nano/microparticles. 0.1996 g copper(II) acetate monohydrate, 0.5984 g stearic acid, 1.3560 g 1-octadecanol, and 25.0760 g 1-octadecene were put into a three-neck round-bottom flask. This flask was placed on a heating mantle with magnetic stirrer. The glass cover was dipped into 1-octadecene and its position was fixed. The glass cover was gently pressed against the flask wall by using a borosilicate glass rod, and thus held steady during the synthesis. A glass reflux condenser was inserted into the middle neck of the flask. A glass coated thermocouple was immersed into 1-octadecene passing through one of the side necks to be able to monitor the real temperature of the mixture. High purity argon gas (99.999%) flowed through the flask throughout the synthesis. This mixture was started to be stirred up at ~754 rpm and heated to 150 °C at 50 min. Care was taken not to splash any drop from the mixture to the flask wall while it was stirring vigorously.

**Figure 2:**
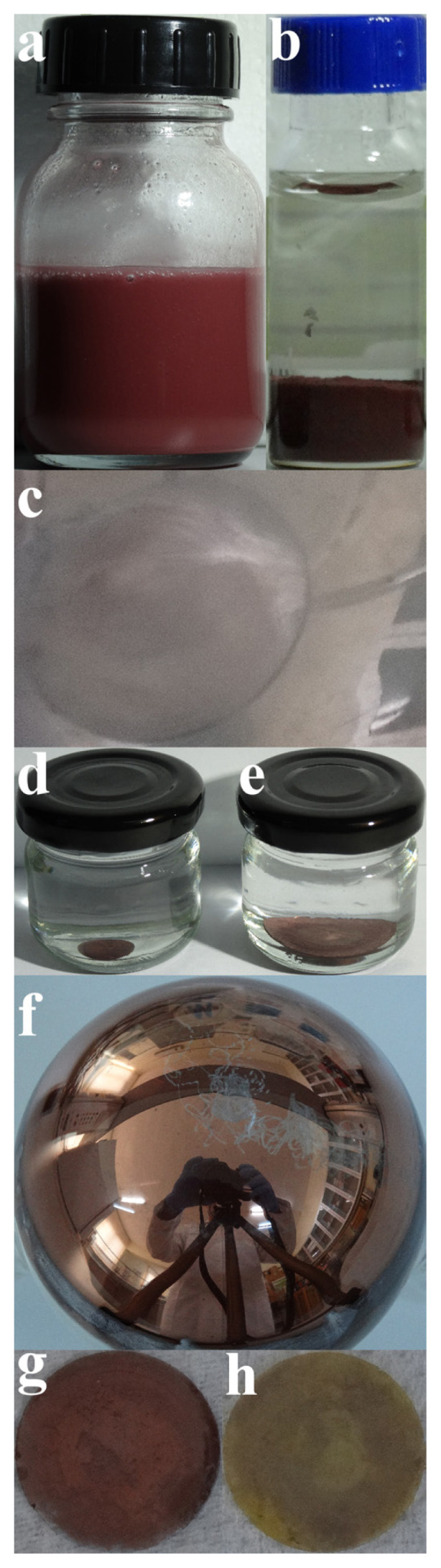
Photographs of samples. a) Copper nano/microparticles synthesized in octadecene, b) Precipitated copper nano/microparticles in toluene, c) Cover glass coated with copper nano/microparticles in flask, d) 10 mm diameter cover glass coated with copper nano/microparticles, which is saved in octadecene against oxidation, e) 24 mm diameter cover glass coated with copper nano/microparticles, which is saved in octadecene against oxidation, f) Copper nano/microparticles-coated flask wall, g) 10 mm diameter cover glass coated with copper nano/microparticles. This sample (shown at g) is called as “Cu n/m-particles on cover glass” in the paper, and h) 10 mm diameter cover glass coated with copper oxide nano/microparticles. This sample (shown at h) is called as “(86% Cu_2_O+14% CuO) n/m-particles on cover glass” in the paper.

The temperature of the mixed solution was raised as a quadratic function of time between 25–150 °C. The colour of the solution turned from green to blue in this temperature range. Then, the temperature was elevated 10 °C per min up to 290 °C and reduced 7 °C per minute until 172 °C. Colourless solution initially appeared at about 231 °C. Yellowish and afterwards reddish-brown colour was observed above 255 °C. The reaction solution was immediately transferred into a glass vial when the temperature was dropped to 172°C (see Figure 2a). Both surfaces of cover glass were coated by copper nano/microparticles during the synthesis (see Figure 2c). The coated cover glass was placed in a glass vial without touching its flat surfaces and stored in 1-octadecene against any oxidation of copper nano/microparticles (see Figures 2d and 2e). The interior wall of the flask was also coated with copper nano/microparticles during the synthesis (see Figure 2f). The cover glass containing copper nano/microparticles on both surfaces was washed with copious amounts of methanol and toluene. The reaction solution was centrifuged thrice at 2500 rpm for 10 min with methanol and toluene to collect copper nano/microparticles from the solution (see Figure 2b). This experiment explained above was repeated many times and a lot of samples were produced and prepared for further characterisations in this way. Some of the copper nano/microparticle coated glass covers were heat treated in a drying and heating oven (Binder FD 115) at 250°C for 2 h to convert copper nano/microparticles to copper oxide nano/microparticles (see Figures 2g and 2h).

### 2.3. Characterisations

Structural and morphological properties of copper nano/microparticles were examined by using X-ray powder diffraction (XRD), X-ray photoelectron spectroscopy (XPS), Raman spectroscopy, scanning electron microscopy (SEM), transmission electron microscopy (TEM), and attenuated total reflectance-Fourier transform infrared spectroscopy (ATR-FTIR). XRD spectra of the samples were measured by using Rigaku SmartLab or Rigaku Ultima-IV diffractometer. Copper K-alpha (1.54 Å) radiation was used for XRD analysis. XPS measurements were taken by PHI 5000 VersaProbe with monochromatized Al K-alpha (1486.6 eV) radiation. Raman spectra were obtained via Renishaw inVia Raman microscope. The samples were excited with a 633 nm wavelength of HeNe laser. Their SEM photos were taken with FEI Quanta 400F field emission scanning electron microscope at 20 kV acceleration voltage. Energy dispersive X-Ray analysis (EDX) was made during SEM. ATR-FTIR spectra of the samples were recorded between 400–4000 cm^−1^ by Perkin Elmer Spectrum 400. Optical transmittance and photoluminescence (PL) spectra of the samples were acquired with optical measurement systems constructed on a scientific grade optical table. Oriel 74000 Cornerstone 1/8 m monochromator including a grating of 1200 lines/mm and a silicon detector attached to this monochromator were used to detect the intensity of light passing through the sample in the visible and near-infrared range of 400–900 nm (corresponding to ~1.38–3.10 eV). The samples were excited with a wavelength of 442 nm of HeCd laser at PL measurement. PL of the samples was collected and focused into the entrance slit of Newport MS257 ¼ m imaging spectrograph with a charge-coupled device detector. The PL spectra were recorded between 450–770 nm (corresponding to ~1.61–2.76 eV). Thermogravimetric (TGA) analysis of some precursors was made via Mettler Toledo TGA/DSC 3+. The temperature of the precursor was raised 10 °C (10 K) per minute under 40 mL/min nitrogen flow between 25–655°C. TEM photos of the sample which was produced from centrifugation of reaction liquid were taken with JEOL JEM-2100F. JEOL JEM-2100F was operated at an acceleration voltage of 200 kV. The average thickness of coatings composed of copper or copper oxide nano/microparticles on glass was measured by Filmetrics Profilm3D optical profilometer.

## 3. Results and discussion

The reaction mechanism can be understood by tracing the colour change of the reaction solution. The solution has acquired blue colour when the temperature increases from 25 °C to 150 °C. As the temperature increases, copper acetate monohydrate initially loses its water (see supplementary Figure S1). Then, copper acetate leaves its acetate anion and gets stearate anion of stearic acid. Thereby copper stearate occurs [24] and it gives blue colour to the solution. The chemical reaction between copper acetate monohydrate and stearic acid can be explained by Equation (1).


(1) $$\text{Cu}{\left({\text{CO}}_{2}{\text{CH}}_{3}\right)}_{2}{\text{H}}_{2}\text{O}+2{\text{CH}}_{3}{\left({\text{CH}}_{2}\right)}_{16}\text{COOH}\to \text{Cu}{\left({\text{CO}}_{2}{\text{CH}}_{3}{\left({\text{CH}}_{2}\right)}_{16}\right)}_{2}+2{\text{CH}}_{3}\text{COOH}+{\text{H}}_{2}\text{O}$$

In Equation (1), Cu(CO_2_CH_3_(CH_2_)_16_)_2_, CH_3_COOH and H_2_O are copper stearate, acetic acid and water products, respectively. The colour of the solution has turned from homogeneous blue to transparent at about 231 °C. It means that copper stearate is thermally decomposed by the help of 1-octadecanol. Thermal decomposition of copper stearate between 200–300 °C under N_2_ gas was investigated by Yuan et al. [25]. They showed that the main mass loss of copper stearate in this temperature range was due to releasing of stearic acid. After this thermal decomposition, the colour of the solution has changed from clear to reddish-brown above 255 °C and the solution has reddish-brown colour at about 264 °C. This colour change is an indicator of the copper particles nucleated and growth in the solution. The oxidation of copper nanoparticles is generally an important problem [26]. The synthesis of copper particles has been done under argon gas for this reason.

**Figure 3:**
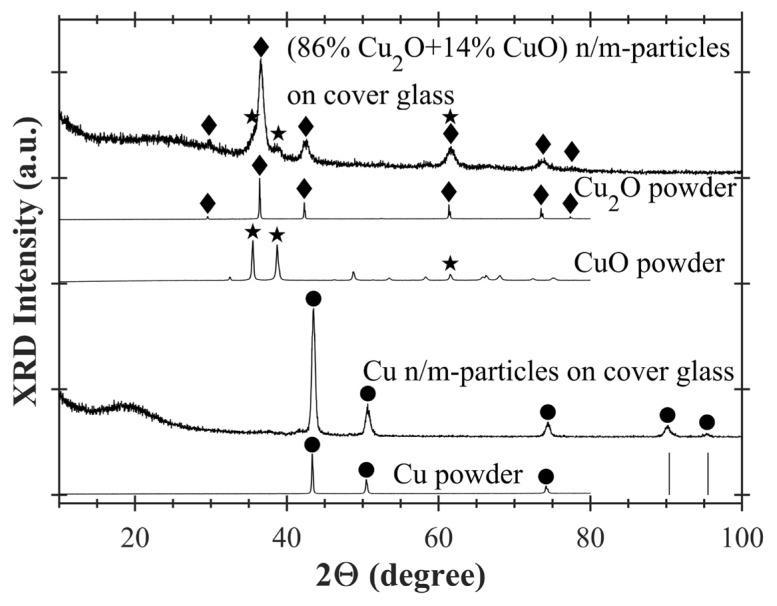
XRD spectra of copper and copper oxide nano/microparticles on cover glass. Black dots (●), stars (★) and diamonds (♦) show XRD peak positions of Cu, CuO, and Cu_2_O, respectively. Vertical lines (|) show copper XRD peak positions. Vertical line positions were taken from reference [27].

XRD spectra of five samples are shown in Figure 3. Three of the samples were bought from Merck. These are powders of copper, copper(II) oxide, and copper(I) oxide. XRD spectra of these powders are labeled as “Cu powder”, “CuO powder”, and “Cu_2_O powder”, respectively, in Figure 3. The remaining two samples are the samples obtained via the synthesis explained above. These synthesis samples are copper nano/microparticles deposited on the cover glass (see Figure 2g) and copper oxide nano/microparticles formed on the cover glass by the heat-treatment (see Figure 2h). XRD spectra of these synthesis samples are labeled as “Cu n/m-particles on cover glass” and “(86% Cu_2_O+14% CuO) n/m-particles on cover glass”, respectively, in Figure 3. Here, XRD spectra of Cu, CuO, and Cu_2_O powders are used as references to identify phases in the synthesis samples. XRD peaks of Cu n/m-particles on cover glass correspond to those of Cu powder between 0–100°. Therefore, it has been understood that sample Cu n/m-particles on cover glass is composed of pure copper particles on glass. The copper phase is marked with black dots (●) in this figure. XRD peaks of (86% Cu_2_O+14% CuO) n/m-particles on cover glass are compatible with those of CuO and Cu_2_O powders. Black stars (★) and diamonds (♦) show CuO and Cu_2_O crystalline phases, respectively. When the reference intensity ratio (RIR) method, which is a quantitative phase analysis used for XRD, is applied to the sample called as (86% Cu_2_O+14% CuO) n/m-particles on cover glass, it has been found that this sample consists of 86 wt% Cu_2_O and 14 wt% CuO. For this reason, this sample has been named as (86% Cu_2_O+14% CuO) n/m-particles on cover glass in the figures and in the text. Comparison of XRD spectra of the cover glass and its Cu n/m-particles coated version is shown at supplementary Figure S2. XRD peak of amorphous silicon dioxide (silica) of cover glass appears around 24°. Thus, the wide XRD peaks seen between 18–24° in Figure 3 have been attributed to the cover glass.

**Figure 4:**
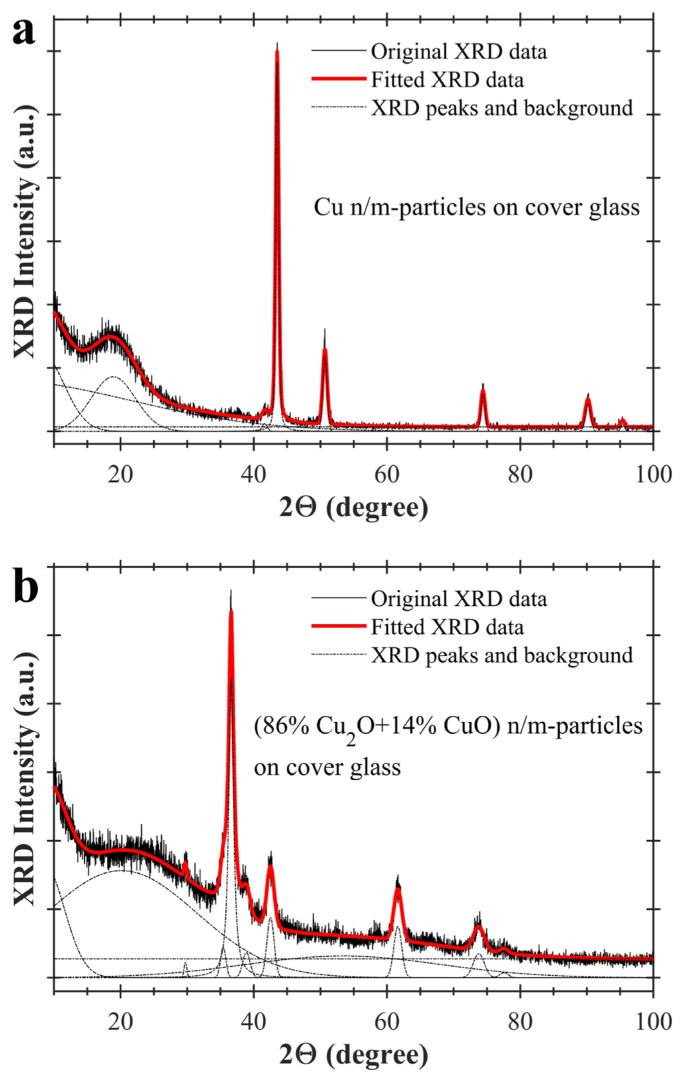
Fitted XRD spectra of copper and copper oxide nano/microparticles on cover glass. a) copper nano/microparticles on cover glass, and b) copper oxide nano/microparticles on cover glass. Gaussian-Lorentzian curves and constant baselines are indicated with dash-dot ( _ . _ . ) lines. Fitted XRD spectra are shown with red colour in the figure.

XRD spectra of the synthesis samples have been fitted by using Gaussian-Lorentzian curves and a constant baseline (background). Used Gaussian-Lorentzian function for the XRD curve-fitting is written at supplementary Equation (S1). Thus, positions, full width at half maxima and relative intensities of XRD peaks have been extracted from the spectra. At Figure 4, XRD spectra of the synthesis samples and their fitted peaks are indicated. XRD spectrum of Cu n/m-particles on cover glass is fitted in Figure 4a and XRD spectrum of (86% Cu_2_O+14% CuO) n/m-particles is fitted in Figure 4b. Fitted curves are drawn with red colour in the figures. Peak positions and full width at half maxima of XRD spectra for two synthesis samples are given in Table 1. 2Θ and β indicate peak position and full width at half maximum (FWHM), respectively, in the table. Grain size (L) for each sample has been determined by Scherrer equation (L = Kλ/(βcosΘ)). As seen in Table 1, average grain sizes have been calculated as ~30.23 nm for Cu n/m-particles and ~13.88 nm for (86% Cu_2_O+14% CuO) n/m-particles. Lattice constant and relative error of lattice constant have also been found for these two samples. Calculated ~0.3% and ~0.06% relative errors confirm copper phase for Cu n/m-particles and copper oxide phase for (86% Cu_2_O+14% CuO) n/m-particles (See Table 1).

**Figure 5:**
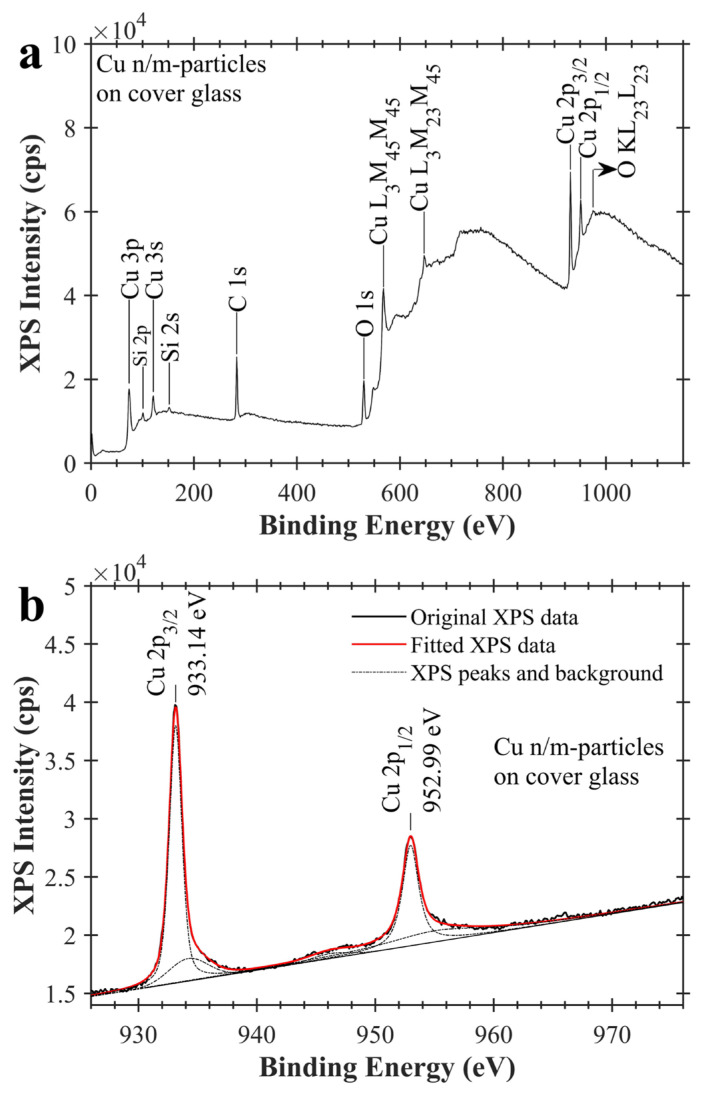
XPS spectra of copper nano/microparticles on cover glass. a) XPS general survey spectrum of Cu n/m-particles on cover glass, and b) XPS scan spectrum of Cu n/m-particles on cover glass between 926–976 eV. Fitted XPS spectrum of Cu n/m-particles on cover glass is shown with red colour. Dash-dot (_ . _ .) lines show Gaussian-Lorentzian and baseline curves which are used for XPS curve-fitting.

XPS spectra of Cu n/m-particles on cover glass are given at Figure 5. XPS peaks of Cu, Si, C, and O have been detected in Figure 5a. Photoelectron and Auger transitions corresponding to these XPS peaks are written in the figure. XPS peaks of Si are due to the bulk of the glass. As seen in Figure 5b, XPS spectrum of the sample was scanned between 926–976 eV. After fitting this XPS spectrum, Cu 2p_3/2_ and Cu 2p_1/2_ XPS peaks have been observed at 933.14 eV and 952.99 eV, respectively. This finding is compatible with XPS data of the literature (See Table 2) [28,29]. Strong shake-up satellite peaks have not seen in two binding energy ranges of 938–946 eV and 958–966 eV for this sample [30–34]. This indicates that there is no CuO in the sample.

**Figure 6:**
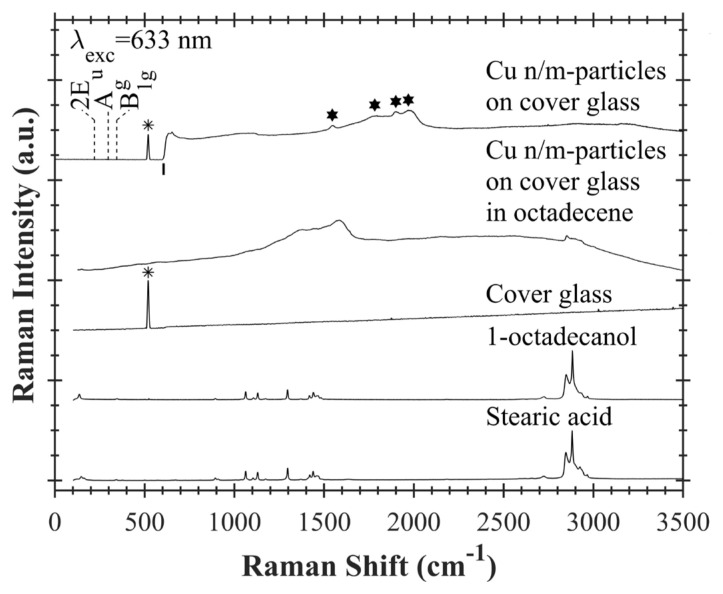
Raman spectra of stearic acid powder, 1-octadecanol powder, cover glass, copper nano/microparticles on cover glass in octadecene and copper nano/microparticles on cover glass. All samples are excited at 633 nm wavelength. Stars (✵) show Si Raman peaks of cover glass. Hexagons (★) designate PL peak positions of Cu n/m-particles on cover glass. 2E_u_, A_g,_ and B_1g_ are Raman modes of copper oxide, and they are not observed in the spectrum. Position of vertical line (|) corresponds to 606 cm^−1^.

In Figure 6, Raman spectra of stearic acid, 1-octadecanol, cover glass, Cu n/m-particles on cover glass in octadecene and Cu n/m-particles on cover glass are shown from bottom to top. Raman peak of Si due to the cover glass is seen at 520 cm^−1^. Stars (★) show these Si Raman peaks in the figure. According to the literature, strong A_g_ and B_1g_ Raman phonon modes are observed at ~297 cm^−1^ and ~345 cm^−1^ for CuO and strong 2E_u_ Raman mode is observed at ~220 cm^−1^ for Cu_2_O [35–38]. These Raman modes have not seen for Cu n/m-particles on cover glass. The absence of A_g_, B_1g_ and 2E_u_ Raman modes in the spectrum of Cu n/m-particles on cover glass is other indication of oxide-free copper in this sample. Above 606 cm^−1^, intensity of Raman signal of this sample sharply increases. There is a high probability that PL signal may be coming from the sample above 606 cm^−1^. Hence, its PL will overlap the Raman signal of the sample itself. Some PL peaks are indicated with hexagons (★) in the figure. Based on these measurements, it has been concluded that the synthesized copper nano/microparticles do not contain any oxide phase.

**Figure 7:**
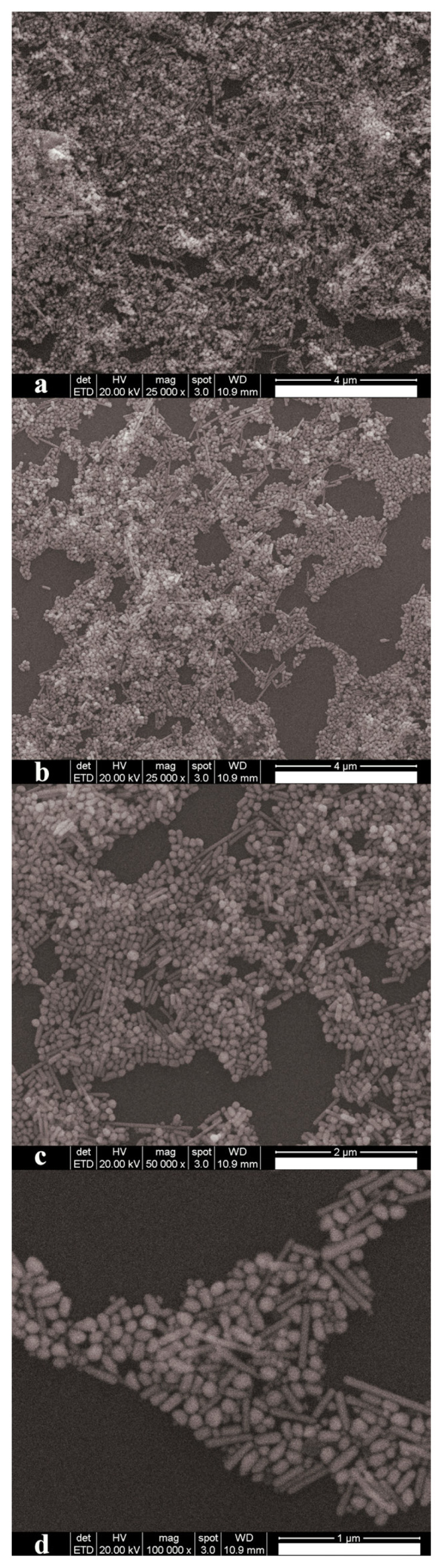
SEM photographs of copper nano/microparticles on cover glass. a) and b) Photos taken at 25,000^X^ magnification and scale bars are 4 Mm, c) Photo taken at 50,000′ magnification and scale bar is 2 Mm, and d) Photo taken at 100,000^X^ magnification and scale bar is 1 Mm.

**Figure 8:**
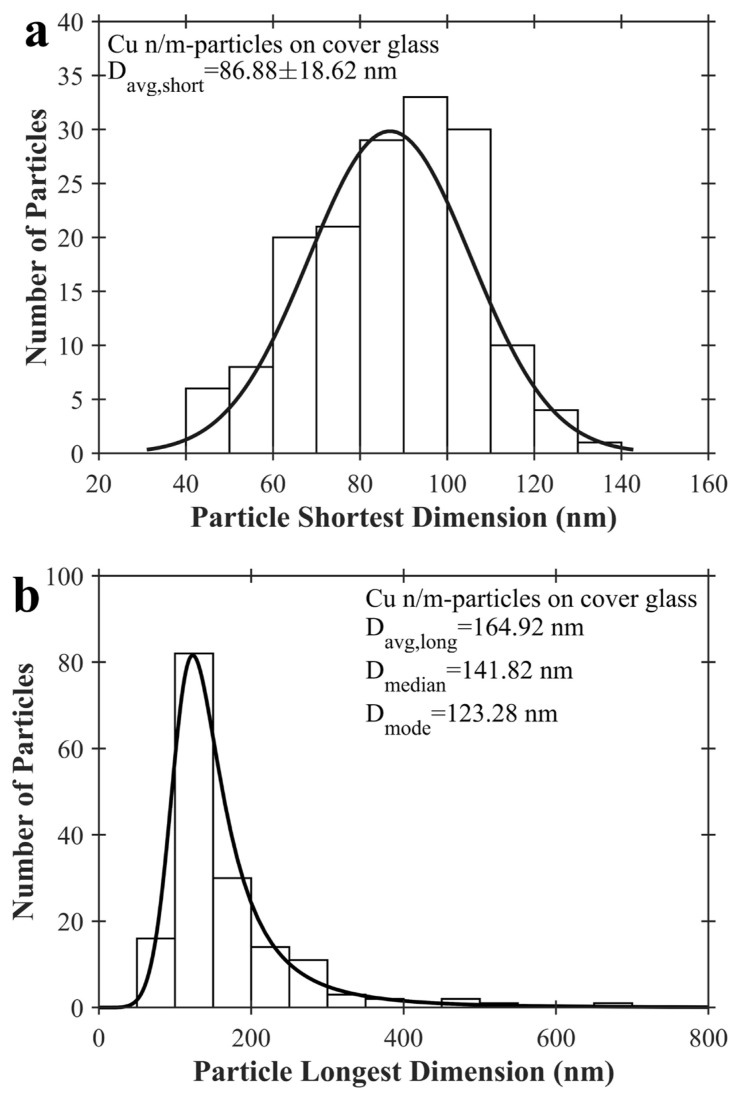
Number of particles versus particle size. a) Versus particle shortest dimension and b) Versus particle longest dimension. D_avg,short_, D_avg,long_, D_median,_ and D_mode_ are average particle size derived by considering shortest length of particles (minimum Feret’s diameter), average particle size derived by considering longest length of particles (maximum Feret’s diameter), median particle size and mode particle size, respectively. Black curves correspond to best curve fits. These are Gaussian and Burr type XII curves for a) and b), respectively. Copper nano/microparticles seen at Figure 7d have been counted and examined to plot Figures 8a and 8b.

SEM photos of Cu n/m-particles on cover glass are shown in Figure 7. These SEM photos were taken at between 25,000^X^–100,000^X^ magnification. In Figure 7a, it is seen that the cover glass is covered by copper particles. Cu nano/microparticles are clearly observed on the cover glass when it is zoomed into the sample from Figure 7a to Figure 7d. As understood from the SEM photos, the sample contains round and nonround nano and micron sized particles, such as rods. Copper seeds in solution growing equally in all directions lead to round particles. However, stearic acid and octadecanol molecules can induce different growth rates along the crystal facets of copper seeds. This might be the reason why some copper particles are rod-shaped. It has been looked at shortest and longest dimensions of the particles via image processing because of the particle shape diversity in the sample. These sizes are known as minimum and maximum Feret’s diameters. The overlapped particles and the particles located at the edges of the image have not been considered during this image processing. One hundred and sixty-two particles have been examined in Figure 7d. Number of particles versus their shortest and longest size has been plotted in Figure 8a and 8b, respectively. Best distribution describing particle size distribution is also shown in these figures. Black curve shows particle size distribution in Figure 8a and 8b. The size distribution is Gaussian (normal) and Burr type XII in Figure 8a and 8b, respectively. The equations of these distributions are written at supplementary Equations (S2) and (S3). Median and average particle sizes for the particle shortest dimension will be same due to the normal distribution and it has been determined to be ~87 ± 19 nm. Moreover, median particle size (it means 50% of the particle sizes is below of this value) considering longest dimension has been found to be ~142 nm. SEM-EDX elemental analysis of Cu n/m-particles on cover glass is shown in supplementary Figure S3. The particle sizes of copper nano/microparticles are greater than their grain sizes. This shows that synthesized copper nano/microparticles have polycrystalline structure.

**Figure 9:**
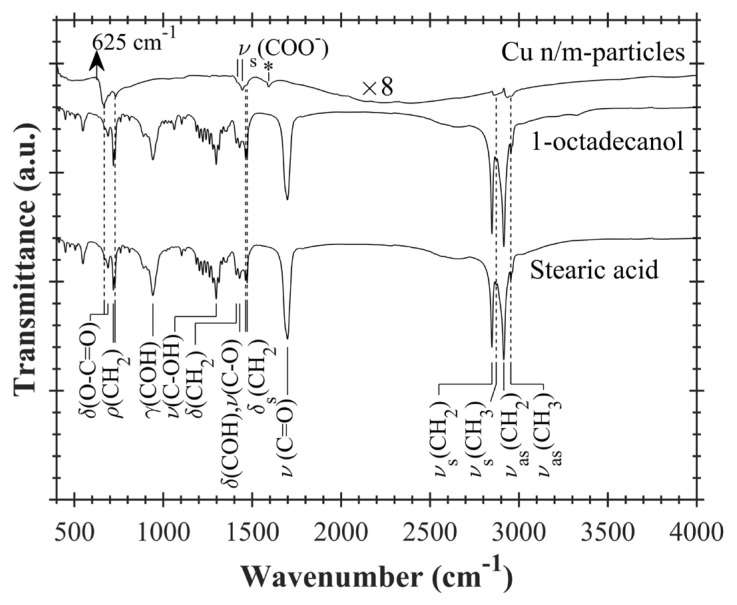
ATR-FTIR transmittance spectra of stearic acid powder, 1-octadecanol powder, and copper nano/microparticles. Transmittance spectrum of copper nano/microparticles is multiplied by 8 (^x^8). ν_s_, ν_as_, ν, δ_s_, δ, ρ, and r indicate symmetric stretching, asymmetric stretching, stretching, scissoring, bending, out of plane bending, and rocking vibrational modes of functional groups such as CH_3_, CH_2_, C=O etc. Star (✵) shows asymmetric vibration mode of COO^−^.

Surface caps of Cu n/m-particles have been investigated by ATR-FTIR spectroscopy. ATR-FTIR transmittance spectra of stearic acid, 1-octadecanol and Cu n/m-particles are shown in Figure 9. The transmittance spectrum of Cu n/m-particles was 8 times intensified by multiplying it with 8 for a better comparison. Vibrational modes of stearic acid and 1-octadecanol are indicated in the figure by using the literature FTIR data [39–41]. Symmetric and antisymmetric vibrations of methyl groups (ν_s_(CH_3_) and ν_as_(CH_3_)), scissoring bending vibration of methylene group (δ_s_(CH_2_)), rocking vibration of methylene group (ρ(CH_2_)), and bending vibration of COO (δ(O-C=O)) have been seen at the spectrum of Cu n/m-particles (See Table 3). In addition, symmetric vibration of COO^−^ (ν_s_(COO^−^)) has also been ascertained. However, strong vibrational modes of stearic acid and 1-octadecanol such as symmetric and antisymmetric vibrations of methylene groups (ν_s_(CH_2_) and ν_as_(CH_2_)) and stretching vibration of CO (ρ(C=O)) have not been observed in the spectrum of Cu n/m-particles. Therefore, it may be inferred that Cu n/m-particles are weakly surrounded by remnant organic molecules after the thermal decomposition. A TEM photo of a Cu n/m-particle precipitated from the reaction solution has been taken (See Figure S4). Surface cap layer on this particle has not been seen. This observation supports the assumption of weak capping. Strong FTIR peaks for CuO and Cu_2_O particles have been reported at around 530 cm^−1^ and 623 cm^−1^, respectively [42–47]. As expected, such peaks have not seen in the spectrum of Cu n/m-particles. Average film thicknesses of Cu n/m-particles and (86% Cu_2_O+14% CuO) n/m-particles have been measured from glass level as ~421.3 nm and ~384.4 nm, respectively (See supplementary Figure S5). The surface roughness of both films has been found to be ~70 nm.

**Figure 10:**
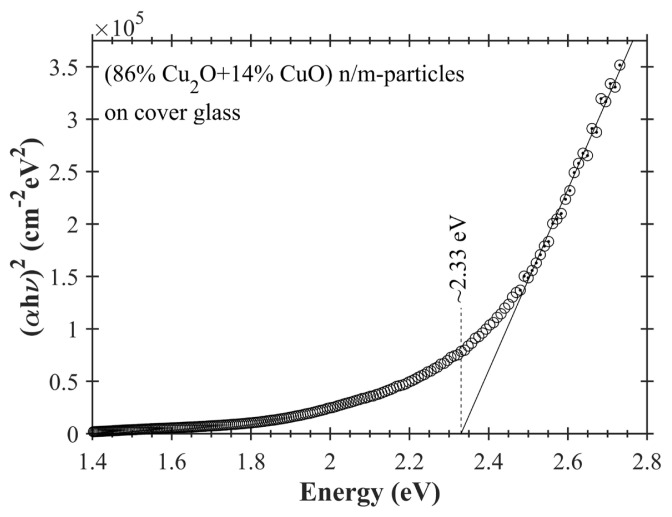
(αhν)^2^ versus photon energy plot (Tauc’s plot) for copper oxide nano/microparticles deposited on cover glass. α is absorption coefficient and hν is photon energy. Circles (0) show measurement data. Solid line indicates linear extrapolation.

Forbidden energy gap of (86% Cu_2_O+14% CuO) n/m-particles on cover glass has been determined by using Tauc’s method [48]. Tauc’s plot is shown for (86% Cu_2_O+14% CuO) n/m-particles in Figure 10. Herein, optical absorption of cover glass has been subtracted from optical absorption of (86% Cu_2_O+14% CuO) n/m-particles on cover glass and so merely optical absorption from copper oxide nano/microparticles has been considered. Accepting that n value equals ½ due to the direct transition for copper oxide [49], (αhn)^1/n^ is plotted against photon energy hν and then linear section of the curve is extrapolated. Interception point of the extrapolated line with hn axis gives the forbidden energy gap. The energy gap of (86% Cu_2_O+14% CuO) n/m-particles has been measured ~2.33 eV by this method. This value is acceptable because the energy gaps of CuO and Cu_2_O (determined from Tauc’s plot) range between ~1.31–2.00 eV and ~1.98–2.58 eV, respectively, in the literature [50–56].

**Figure 11:**
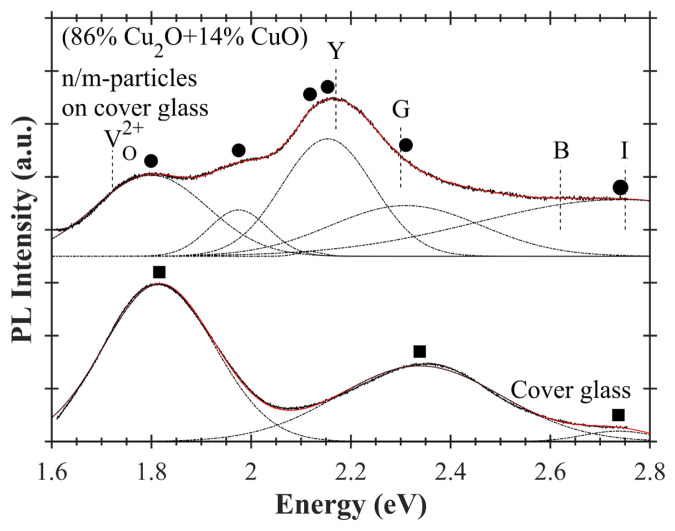
PL spectra of cover glass and copper oxide nano/microparticles on cover glass. PL peak positions are determined by curve fitting. Gaussian curves are used for curve fitting of PL spectra. Dash-dot curves (_ . _ .) show these Gaussian curves. Red curves show fitted PL spectra. PL peaks of cover glass are indicated by black squares (■). Black dots (●) indicate PL peaks of (86% Cu_2_O+14% CuO) n/m-particles on cover glass. Y, G, B, and I correspond to PL peaks arising from yellow, green, blue, and indigo excitonic transitions of Cu_2_O, respectively. ${\text{V}}_{\text{O}}^{2+}$ points out PL peak position of doubly charged oxygen vacancy of Cu_2_O.

PL measurements of cover glass and (86% Cu_2_O+14% CuO) n/m-particles on cover glass are shown in Figure 11. In this figure, PL spectrum of cover glass is given below and PL spectrum of (86% Cu_2_O+14% CuO) n/m-particles on cover glass is given above. Gaussian peaks have been used for curve fittings of PL spectra. Black squares (■) and black dots (●) indicate these Gaussian peak positions. In other words, they correspond to PL peak positions. Red lines show the curve fittings. PL peak positions of cover glass have been found at ~1.82 eV, ~2.34 eV, and ~2.74 eV (See Table 4). At this point, it should be stated that a soda-lime glass was excited with 488 nm at 77 K by Martínez-Saucedo et al. [51] and they observed PL peaks of a soda-lime glass at energies close to those of the cover glass. Six peak positions have been determined for (86% Cu_2_O+14% CuO) n/m-particles on cover glass. These peaks are at ~1.80 eV, ~1.98 eV, ~2.12 eV, ~2.15 eV, ~2.31 eV, and ~2.74 eV. According to the electronic band structure of Cu_2_O, band gap PL ( $\Gamma_{6}^{+}\to \Gamma_{7}^{+}$ transition) appears at ~2.17 eV and excitonic transitions of Cu_2_O due to yellow, green, blue, and indigo series are in the range of ~2.17–2.75 eV [57,58]. The PL peak at ~2.15 eV has located close to the transitions of yellow series excitons, and it is slightly below the optical energy gap of ~2.33 eV. For these reasons, it can be accepted as room temperature band gap PL of (86% Cu_2_O+14% CuO) n/m-particles.

## 4. Conclusions

Copper nano/microparticles have been formed and growth in noncoordinating solvent octadecene by using one-pot thermal decomposition method. Borosilicate cover glasses have been immersed in octadecene during the synthesis for coating them with copper nano/microparticles. This could be a new approach to thermal decomposition because coating of cover glass with copper nano/microparticles occurs concurrently with thermal decomposition. Copper(II) acetate monohydrate and stearic acid have been reacted to form copper stearate complex and then this complex has been thermally decomposed into copper nano/microparticles at 290 °C under argon gas. 1-octadecanol has been used as a mild reducer in the synthesis. Unlike other thermal decomposition syntheses, copper acetate monohydrate, stearic acid, and 1-octadecanol have been used in one-pot. A thoroughly characterisation has been done for copper nano/microparticles coated cover glasses. Pure copper crystalline phase of nano/microparticles has been revealed by means of XRD, XPS, Raman, SEM-EDX, and ATR-FTIR measurements. Median copper particle size has been found as ~87 ± 19 nm for shortest length and ~142 nm for longest length by examining Feret’s diameters of the particles with SEM, respectively. SEM images have showed that synthesized copper particles are consists of nano and micron sized particles. Weakly capped surface of copper nano/microparticles with organic molecules arising from thermal decomposition has been indicated by ATR-FTIR and TEM. Copper nano/microparticles have been converted into copper oxide nano/microparticles after heat treatment at 250 °C in air atmosphere. Both CuO and Cu_2_O phases have been observed at XRD diffractogram of copper oxide nano/microparticles. Percent weight composition of copper oxide nano/microparticles has been evaluated by XRD-RIR analysis. It has been found that copper oxide nano/microparticles are composed of 14wt% CuO and 86wt% Cu_2_O. Average thickness of copper oxide film layer has been measured as ~384.4 nm by optical profilometer. Energy gap of these copper oxide nano/microparticles have been determined as ~2.33 eV by means of Tauc’s plot method. PL emission slightly below the energy gap of copper oxide nano/microparticles has been observed at ~2.15 eV. After the characterisations explained above, antibacterial properties of these nano/microparticles will be studied in future.

## Supplement

First remarkable mass loss range is observed at between ~126–159 °C due to releasing of water from copper(II) acetate monohydrate (see Figure S1). This temperature range has been determined by the extrapolation method.

XRD peak of amorphous SiO_2_ has been appeared at ~24° (see Figure S2).

Surface morphologies of films of copper and copper oxide nanoparticles are shown at Figure S5.


(S1) $$I_{G-L}(2\theta )=a_{0}\;\left[\frac{\frac{a_{3}\sqrt{Ln2}}{a_{2}\sqrt{\pi }}e^{-4(Ln2){\left(\frac{2\theta -a_{1}}{a_{2}}\right)}^{2}}+\frac{1-a_{3}}{\pi a_{2}\;\left[1+4\;{\left(\frac{2\theta -a_{1}}{a_{2}}\right)}^{2}\right]}}{\frac{a_{3}\sqrt{Ln2}}{a_{2}\sqrt{\pi }}+\frac{1-a_{3}}{\pi a_{2}}}\right]$$

Where 2Θ is position in degrees, I_G-L_(2Θ) is intensity of XRD peak depending on 2Θ, a_0_ is amplitude of XRD peak, a_1_ is center of XRD peak, a_2_ is FWHM of XRD peak, and a_3_ is shape factor of XRD peak. Here, a_2_ > 0 and 0 ≤ a_3_ ≤1.

A Gaussian and Burr type XII functions are given at Equations (S2) and (S3), respectively. Here, these functions represent particle size distributions.


(S2) $$N_{G}(d)=a_{0}e^{-0.5{\left(\frac{d-a_{1}}{a_{2}}\right)}^{2}}$$

Where d is particle size, N_G_(d) is number of particles for any particle size of d in distribution, a_0_ is maximum particle number for d = a_1_, a_1_ is average particle size of particle size distribution and a_2_ is standard deviation of particle size distribution. Here, a_2_ > 0.


(S3) $$N_{B}(d)=\frac{\frac{a_{0}a_{1}}{a_{2}}{\left(\frac{d}{a_{2}}\right)}^{a_{1}-1}}{{\left[1+{\left(\frac{d}{a_{2}}\right)}^{a_{1}}\right]}^{a_{0}+1}}$$

Where d is particle size and N_B_(d) is number of particles for any particle size of d in distribution. In (S3), a_0_, a_1,_ and a_2_ are parameters which define mean value and standard deviation. Here, a_0_ > 0, a_1_ > 0, and a_2_ > 0.

Figure S1 TGA curve of copper(II) acetate monohydrate. 12.5580 mg copper(II) acetate monohydrate was analyzed. Temperature of this sample was increased from ~24 °C to ~654 °C. Heating rate was 10 °C/min in N_2_ gas atmosphere. N_2_ gas flow rate was 40 mL/min.

Figure S2 XRD spectra of cover glass and copper nano/microparticles (Cu n/m-particles) on cover glass. Black squares (■) indicate amorphous SiO_2_ XRD peak of cover glass. Black dots (●) show pure copper XRD peaks of nano/microparticles.

Figure S3 SEM-EDX spectrum of copper nano/microparticles on cover glass. Cu peaks are seen.

Figure S4 TEM photos of a copper nano/microparticle. a) Scale bar is 100 nm, and b) Scale bar is 50 nm. This copper nano/microparticle was precipitated from the reaction solution by centrifugation process.

Figure S5 Film thicknesses of copper and copper oxide nano/microparticles on cover glass. a) Film thickness of Cu n/m-particles, and b) Film thickness of (86% Cu_2_O+14% CuO) n/m-particles. Vertical colour bars given at the left show film heights and each colour corresponds to a different height. Surface morphologies of these deposited films (coatings) were measured by using Filmetrics Profilm3D optical profilometer.
A Gaussian-Lorentzian function is described at Equation (S1). Here, this equation represents a XRD peak in the context of this paper.

## Figures and Tables

**Table 1:** Results of XRD measurements of Cu n/m-particles and (86% Cu_2_O+14% CuO) n/m-particles. In the first column, K,λ, β, 2Θ, L, L_avg_, d, (h,k,l), a, a_avg_, a_bulk_ and relative error of a are shape factor, wavelength of X-ray, full width at half maximum, double Bragg angle, grain size, average grain size, distance between adjacent crystal planes, Miller indices, lattice constant, average lattice constant, lattice constant of bulk material, relative error of lattice constant, respectively. Second and third columns show the XRD results for Cu n/m particles and (86% Cu_2_O+14% CuO) n/m-particles, respectively.

Derived Parameters from XRD	Cu n/m-particles	(86%Cu_2_O+14%CuO) n/m-particles
K	0.94000	0.94000
λ[nm]	0.15418	0.15418
β[^o^]	0.53274 0.32684 0.30769 0.38667 0.29413	0.86934 0.53884 0.59627 0.81751
2q[^o^]	43.51440 50.67771 74.40282 90.16914 95.38012	36.60162 42.51246 61.62324 73.77617
L[nm] =Kλ/(βcosΘ) for b in radians	16.78257 28.11089 33.88217 30.41550 41.94047	10.06074 16.53551 16.21493 12.69986
L_avg_[nm]	30.22632	13.87776
d[nm] =λ/(2sinΘ) for n = 1	0.20797 0.18013 0.12750 0.10886 0.10424	0.24551 0.21264 0.15050 0.12843
(h,k,l)	(1,1,1) (2,0,0) (2,2,0) (3,1,1) (2,2,2)	(1,1,1) (2,0,0) (2,2,0) (3,1,1)
a[nm] = d(h^2^+k^2^+l^2^)^1/2^ for cubic unit cell	0.36022 0.36026 0.36063 0.36105 0.36111	0.42523 0.42528 0.42569 0.42595
a_avg_[nm]	0.36065	0.42554
a_bulk_[nm]	0.36150	0.42580*
Relative error of a (%)	~0.3%	~0.06%

* 0.42580 nm is lattice constant of bulk Cu_2_O.

**Table 2:** Results of XPS measurement of Cu n/m-particles. Cu 2p_1/2_ and Cu 2p_3/2_ photoelectron, and Cu L_3_M_23_M_45_ and CuL_3_M_45_M_45_ Auger transitions of Cu n/m-particles are listed. XPS data from literature are given for comparison in the third column.

Derived parameters from XPS	Cu n/m-particles	Literature XPS data*
Cu 2p_1/2_ [eV]	952.9869	952.45
Cu 2p_3/2_ [eV]	933.1440	932.67
Cu L_3_M_23_M_45_ [eV]	647	647.59
Cu L_3_M_45_M_45_ [eV]	568	568.11

* See references [28,29].

**Table 3:** Results of ATR-FTIR measurements of Cu n/m-particles and stearic acid. Infrared vibrational modes of Cu n/m-particles are compared with those of stearic acid and literature FTIR data.

Derived parameters from FTIR*	Cu n/m-particles	Stearic acid	Literature FTIR data**
ν_as_(CH_3_) [cm^−1^]	2957	2954	2953, 2961
ν_s_(CH_3_) [cm^−1^]	2859	2872	2871
δ_s_(CH_2_) [cm^−1^]	1460, 1468	1463, 1472	1462, 1467, 1471
ν_s_(COO^−^) [cm^−1^]	1418, 1445	-	1397, 1399–1444
ρ(CH_2_) [cm^−1^]	731	719, 729	719, 728, 720–722
δ(O-C=O) [cm^−1^]	668, 696	668, 689	670, 688, 636–711

* Vibrational modes. ν_s_: symmetric stretching, ν_as_: asymmetric stretching, δ: bending, δ_s_: scissoring, ρ: rocking.

** See references [39–41].

**Table 4:** PL peak positions of (86% Cu_2_O+14% CuO) n/m-particles and cover glass.

Derived parameters from PL	(86%Cu_2_O+ 14%CuO) n/m-particles	Cover glass
Peak positions [eV]	1.799 1.975 2.118 2.154 2.310 2.741	1.816 2.338 2.736

## References

[1] Kim D Kley CS Li Y Yang P Copper nanoparticle ensembles for selective electroreduction of CO_2_ to C_2_-C_3_ products PNAS 2017 114 40 10560 10565 10.1073/pnas.1711493114 28923930 PMC5635920

[2] Chen D Zhu S Li W Kang Z Stable superhydrophobic and conductive surface: fabrication of interstitial coral-like copper nanostructure by self-assembly and spray deposition Colloids and Surfaces A: Physicochemical and Engineering Aspects 2022 638 1 10 10.1016/j.colsurfa.2022.128299

[3] Hong S Liu C Hao S Fu W Peng J Antioxidant high-conductivity copper paste for low-cost flexible printed electronics npj Flexible Electronics 2022 6 1 9 10.1038/s41528-022-00151-1

[4] Kang S Tasaka K Lee JH Yabuki A Self-reducible copper complex inks with two amines for copper conductive films via calcination below 100°C Chemical Physics Letters 2021 763 1 6 10.1016/j.cplett.2020.138248

[5] Grass G Rensing C Solioz M Metallic copper as an antimicrobial surface Applied and Environmental Microbiology 2011 77 5 1541 1547 10.1128/AEM.02766-10 21193661 PMC3067274

[6] Hutasoit N Kennedy B Hamilton S Luttick A Rashid RAR Sars-CoV-2 (COVID-19) inactivation capability of copper-coated touch surface fabricated by cold-spray technology Manufacturing Letters 2020 25 93 97 10.1016/j.mfglet.2020.08.007 32904558 PMC7455544

[7] Park J Kwon T Kim J Jin H Kim HY Hollow nanoparticles as emerging electrocatalysts for renewable energy conversion reactions Chemical Society Reviews 2018 47 22 8173 8202 10.1039/C8CS00336J 30009297

[8] Tauran Y Brioude A Coleman AW Rhimi M Kim B Molecular recognition by gold, silver and copper nanoparticles World Journal of Biological Chemistry 2013 4 3 35 63 10.4331/wjbc.v4.i3.35 23977421 PMC3746278

[9] Effenberger FB Sulca MA Machini MT Couto RA Kiyohara PK Copper nanoparticles synthesized by thermal decomposition in liquid phase: the influence of capping ligands on the synthesis and bactericidal activity Journal of Nanoparticle Research 2014 16 1 10 10.1007/s11051-014-2588-7

[10] Park J An K Hwang Y Park J-G Noh H-J Ultra-large-scale syntheses of monodisperse nanocrystals Nature Materials 2004 3 891 895 10.1038/nmat1251 15568032

[11] Betancourt-Galindo R Reyes-Rodriguez PY Puente-Urbina BA Avila-Orta CA Rodríguez-Fernández OS Synthesis of copper nanoparticles by thermal decomposition and their antimicrobial properties Journal of Nanomaterials 2014 2014 1 5 10.1155/2014/980545

[12] Devaraj M Saravanan R Deivasigamani RK Gupta VK Gracia F Fabrication of novel shape Cu and Cu/Cu_2_O nanoparticles modified electrode for the determination of dopamine and paracetamol Journal of Molecular Liquids 2016 221 930 941 10.1016/j.molliq.2016.06.028

[13] Kang YS Kim YH Jo BG Jeong JH Synthesis and characterization of Cu nanoparticles prepared by thermal decomposition of Cu-oleate complex International Journal of Nanoscience 2006 5 2–3 339 344 10.1142/S0219581X06004449

[14] Diab M Moshofsky B Plante IJ-L Mokari T A facile one-step approach for the synthesis and assembly of copper and copper-oxide nanocrystals Journal of Materials Chemistry 2011 21 31 11626 11630 10.1039/C1JM10638D

[15] Salavati-Niasari M Davar F Mir N Synthesis and characterization of metallic copper nanoparticles via thermal decomposition Polyhedron 2008 27 17 3514 3518 10.1016/j.poly.2008.08.020

[16] Togashi T Nakayama M Hashimoto A Ishizaki M Kanaizuka K Solvent-free synthesis of monodisperse Cu nanoparticles by thermal decomposition of an oleylamine-coordinated Cu oxalate complex Dalton Transactions 2018 47 15 5342 5347 10.1039/C8DT00345A 29589610

[17] Son SU Park IK Park J Hyeon T Synthesis of Cu_2_O coated Cu nanoparticles and their successful applications to Ullmann-type amination coupling reactions of aryl chlorides Chemical Communications 2004 7 778 779 10.1039/B316147A 15045059

[18] Wei Y Chen S Kowalczyk B Huda S Gray TP Synthesis of stable, low-dispersity copper nanoparticles and nanorods and their antifungal and catalytic properties Journal of Physical Chemistry C 2010 114 37 15612 15616 10.1021/jp1055683

[19] Salavati-Niasari M Davar F Synthesis of copper and copper(I) oxide nanoparticles by thermal decomposition of a new precursor Materials Letters 2009 63 3–4 441 443 10.1016/j.matlet.2008.11.023

[20] Togashi T Nakayama M Miyake R Uruma K Kanaizuka K N,N-diethyl-diaminopropane-copper(II) oxalate self-reducible complex for the solution-based synthesis of copper nanocrystals Dalton Transactions 2017 46 37 12487 12493 10.1039/C7DT02510F 28895601

[21] Qu B Lu X Wu Y You X Xu X Synthesis of copper micro-rods with layered nano-structure by thermal decomposition of the coordination complex Cu(BTA)_2_ Nanoscale Research Letters 2015 10 42 1 5 10.1186/s11671-015-0769-7 25852339 PMC4384981

[22] Adner D Korb M Schulze S Hietschold M Lang H A straightforward approach to oxide-free copper nanoparticles by thermal decomposition of a copper(I) precursor Chemical Communications 2013 49 61 6855 6857 10.1039/C3CC42914H 23792829

[23] Hambrock J Becker R Birkner A Weiß J Fischer RA A non-aqueous organometallic route to highly monodispersed copper nanoparticles using [Cu(OCH(Me)CH_2_NMe_2_)_2_] Chemical Communications 2002 1 68 69 10.1039/B108797E 12120314

[24] Eren GO Sadeghi S Shahzad M Nizamoglu S Protocol on synthesis and characterization of copper-doped InP/ZnSe quantum dots as ecofriendly luminescent solar concentrators with high performance and large area STAR Protocols 2021 2 3 1 18 10.1016/j.xpro.2021.100664 PMC8283155 34308379

[25] Yuan C Varfolomeev MA Emelianov DA Suwaid MA Khachatrian AA Copper stearate as a catalyst for improving the oxidation performance of heavy oil in in-situ combustion process Applied Catalysis A: General 2018 564 79 89 10.1016/j.apcata.2018.07.021

[26] Jardón-Maximino N Pérez-Alvarez M Sierra-Ávila R Ávila-Orta CA Jiménez-Regalado E Oxidation of copper nanoparticles protected with different coatings and stored under ambient conditions Journal of Nanomaterials 2018 2018 1 8 10.1155/2018/9512768

[27] Liu A Shi Z Reddy RG Mechanism study of Cu-Zn alloys electrodeposition in deep eutectic solvents Ionics 2020 26 3161 3172 10.1007/s11581-019-03418-2

[28] Miller AC Simmons GW Copper by XPS Surface Science Spectra 1993 2 1 55 60 10.1116/1.1247725

[29] Raja M Subha J Ali FB Ryu SH Synthesis of copper nanoparticles by electroreduction process Materials and Manufacturing Processes 2008 23 8 782 785 10.1080/10426910802382080

[30] Biesinger MC Advanced analysis of copper X-ray photoelectron spectra Surface and Interface Analysis 2017 49 13 1325 1334 10.1002/sia.6239

[31] Ethiraj AS Kang DJ Synthesis and characterization of CuO nanowires by a simple wet chemical method Nanoscale Research Letters 2012 7 1 5 10.1186/1556-276X-7-70 22221503 PMC3283496

[32] Hesabizadeh T Jebari N Madouri A Hallais G Clark TE Electric-field-induced phase change in copper oxide nanostructures ACS Omega 2021 6 48 33130 33140 10.1021/acsomega.1c05498 34901664 PMC8655937

[33] Hiraba H Koizumi H Kodaira A Nogawa H Yoneyama T Influence of oxidation of copper on shear bond strength to an acrylic resin using an organic sulfur compound Materials 2020 13 9 1 8 10.3390/ma13092092 PMC7254407 32370001

[34] Zatsepin DA Boukhvalov DW Kurmaev EZ Zatsepin AF Kim SS Enhanced clustering tendency of Cu-impurities with a number of oxygen vacancies in heavy carbon-loaded TiO_2_ - the bulk and surface morphologies Solid State Sciences 2017 71 130 138 10.1016/j.solidstatesciences.2017.07.013

[35] Akgul FA Akgul G Yildirim N Unalan HE Turan R Influence of thermal annealing on microstructural, morphological, optical properties and surface electronic structure of copper oxide thin films Materials Chemistry and Physics 2014 147 3 987 995 10.1016/j.matchemphys.2014.06.047

[36] Litvinchuk AP Möller A Debbichi L Krüger P Iliev MN Second-order Raman scattering in CuO Journal of Physics: Condensed Matter 2013 25 10 1 5 10.1088/0953-8984/25/10/105402 23388624

[37] Sander T Reindl CT Klar PJ Breaking of Raman selection rules in Cu_2_O by intrinsic point defects Symposium R-Oxide Semiconductors, MRS Online Proceedings Library 1633 Boston, Massachusetts, USA 2013 81 86 10.1557/opl.2014.47

[38] Shyamal S Hajra P Mandal H Singh JK Satpati AK Effect of substrates on the photoelectrochemical reduction of water over cathodically electrodeposited p-type Cu_2_O thin films ACS Applied Materials & Interfaces 2015 7 33 18344 18352 10.1021/acsami.5b04116 26244558

[39] Dou Q Ng KM Synthesis of various metal stearates and the corresponding monodisperse metal oxide nanoparticles Powder Technology 2016 301 949 958 10.1016/j.powtec.2016.07.037

[40] Filopoulou A Vlachou S Boyatzis SC Fatty acids and their metal salts: a review of their infrared spectra in light of their presence in cultural heritage Molecules 2021 26 19 1 27 10.3390/molecules26196005 PMC8512802 34641549

[41] Pudney PDA Mutch KJ Zhu S Characterising the phase behaviour of stearic acid and its triethanolamine soap and acid–soap by infrared spectroscopy Physical Chemistry Chemical Physics 2009 11 25 5010 5018 10.1039/B819582J 19562130

[42] Dehaj MS Mohiabadi MZ Experimental study of water-based CuO nanofluid flow in heat pipe solar collector Journal of Thermal Analysis and Calorimetry 2019 137 2061 2072 10.1007/s10973-019-08046-6

[43] Khan MA Ullah M Iqbal T Mahmood H Khan AA Surfactant assisted synthesis of cuprous oxide (Cu_2_O) nanoparticles via solvothermal process Nanoscience and Nanotechnology Research 2015 3 1 16 22

[44] Kooti M Matouri L Fabrication of nanosized cuprous oxide using Fehling’s solution Scientia Iranica 2010 17 1 73 78

[45] Luna IZ Hilary LN Chowdhury AMS Gafur MA Khan N Preparation and characterization of copper oxide nanoparticles synthesized via chemical precipitation method Open Access Library Journal 2015 2 3 1 8 10.4236/oalib.1101409

[46] Sahai A Goswami N Kaushik SD Tripathi S Cu/Cu_2_O/CuO nanoparticles: novel synthesis by exploding wire technique and extensive characterization Applied Surface Science 2016 390 974 983 10.1016/j.apsusc.2016.09.005

[47] Tahir M Zeb M Alamgeer Hussain S Sarker MR Cuprous oxide nanoparticles: synthesis, characterization, and their application for enhancing the humidity-sensing properties of poly(dioctylfluorene) Polymers 2022 14 8 1 14 10.3390/polym14081503 PMC9028022 35458255

[48] Makuła P Pacia M Macyk W How to correctly determine the band gap energy of modified semiconductor photocatalysts based on uv-vis spectra Journal of Physical Chemistry Letters 2018 9 23 6814 6817 10.1021/acs.jpclett.8b02892 30990726

[49] Balamurugan B Aruna I Mehta BR Shivaprasad SM Size-dependent conductivity-type inversion in Cu_2_O nanoparticles Physical Review B 2004 69 16 1 5 10.1103/PhysRevB.69.165419

[50] Hssi AA Atourki L Labchir N Ouafi M Abouabassi K High-quality Cu_2_O thin films via electrochemical synthesis under a variable applied potential Journal of Materials Science: Materials in Electronics 2020 31 4237 4244 10.1007/s10854-020-02976-w

[51] Martínez-Saucedo G Torres-Castanedo CG Arias-Cerón S Castanedo-Pérez R Torres-Delgado G Photoluminescence of Cu_2_O nanostructured in stressed thin films induced by temperature Journal of Luminescence 2019 215 1 7 10.1016/j.jlumin.2019.116642

[52] Murali DS Kumar S Choudhary RJ Wadikar AD Jain MK Synthesis of Cu_2_O from CuO thin films: optical and electrical properties AIP Advances 2015 5 4 1 5 10.1063/1.4919323

[53] Sudha V Murugadoss G Thangamuthu R Structural and morphological tuning of Cu-based metal oxide nanoparticles by a facile chemical method and highly electrochemical sensing of sulphite Scientific Reports 2021 11 1 12 10.1038/s41598-021-82741-z 33564014 PMC7873194

[54] Wang Y Miska P Pilloud D Horwat D Mücklich F Transmittance enhancement and optical band gap widening of Cu_2_O thin films after air annealing Journal of Applied Physics 2014 115 7 1 5 10.1063/1.4865957

[55] Yang Y Xu D Wu Q Diao P Cu_2_O/CuO bilayered composite as a high-efficiency photocathode for photoelectrochemical hydrogen evolution reaction Scientific Reports 2016 6 1 13 10.1038/srep35158 27748380 PMC5066255

[56] Zhang L McMillon L McNatt J Gas-dependent bandgap and electrical conductivity of Cu_2_O thin films Solar Energy Materials & Solar Cells 2013 108 230 234 10.1016/j.solmat.2012.05.010

[57] Aßmann M Bayer M Semiconductor Rydberg Physics Advanced Quantum Technologies 2020 3 11 1 20 10.1002/qute.201900134

[58] Ko E Choi J Okamoto K Tak Y Lee J Cu_2_O nanowires in an alumina template: electrochemical conditions for the synthesis and photoluminescence characteristics ChemPhysChem 2006 7 7 1505 1509 10.1002/cphc.200600060 16733843

