# Supplementary figures and images for: Synthesis of copper nano/microparticles via thermal decomposition and their conversion to copper oxide film
Source: Turk J Chem. 2023 May 9;47(3):616–32. doi: 10.55730/1300-0527.3565 (PMC10388133; doi:10.55730/1300-0527.3565)

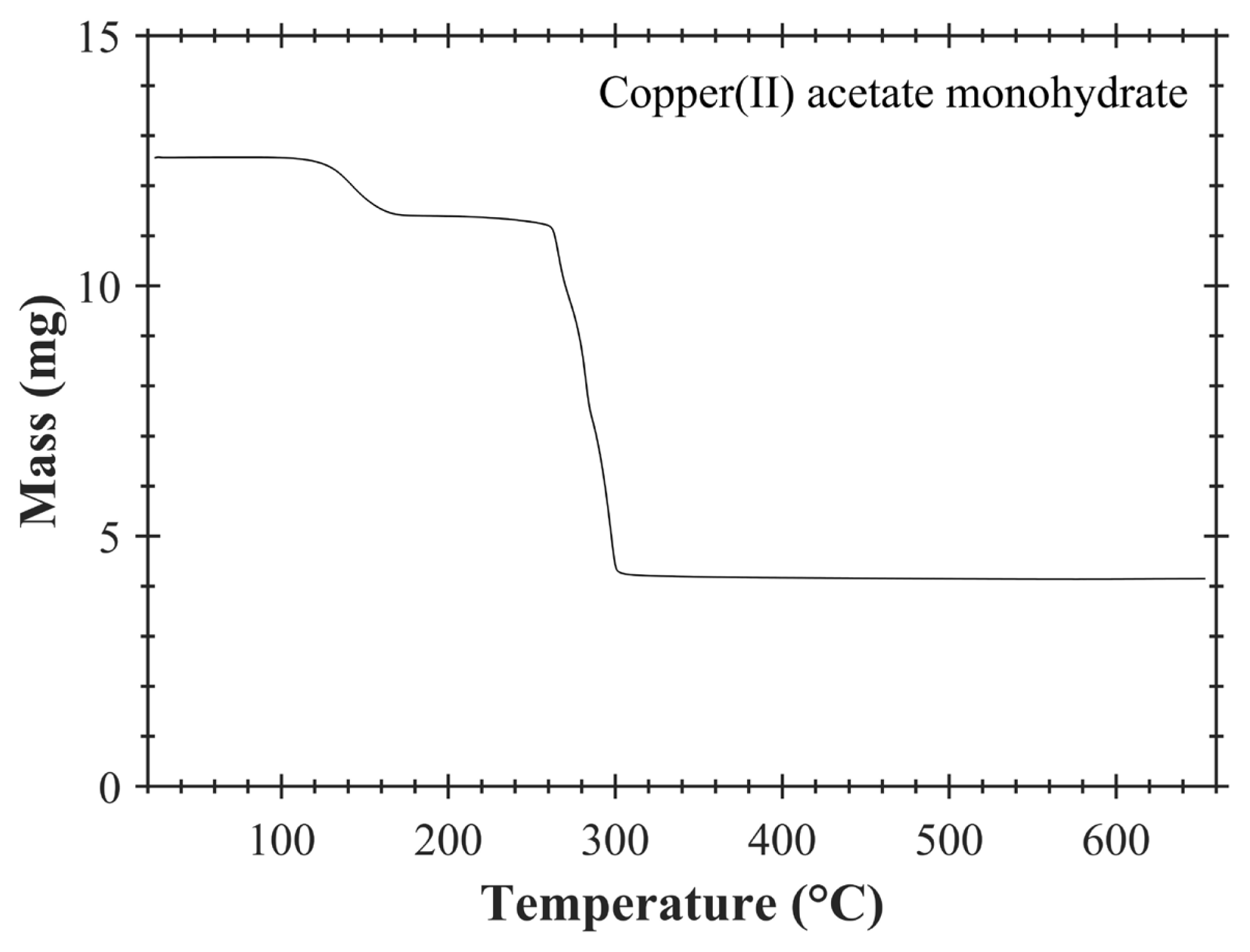

Supplement: Figure S1 — TGA curve of copper(II) acetate monohydrate. 12.5580 mg copper(II) acetate monohydrate was analyzed. Temperature of this sample was increased from ~24 °C to ~654 °C. Heating rate was 10 °C/min in N2 gas atmosphere. N2 gas flow rate was 40 mL/min. [file turkjchem-47-3-616s1.tif]

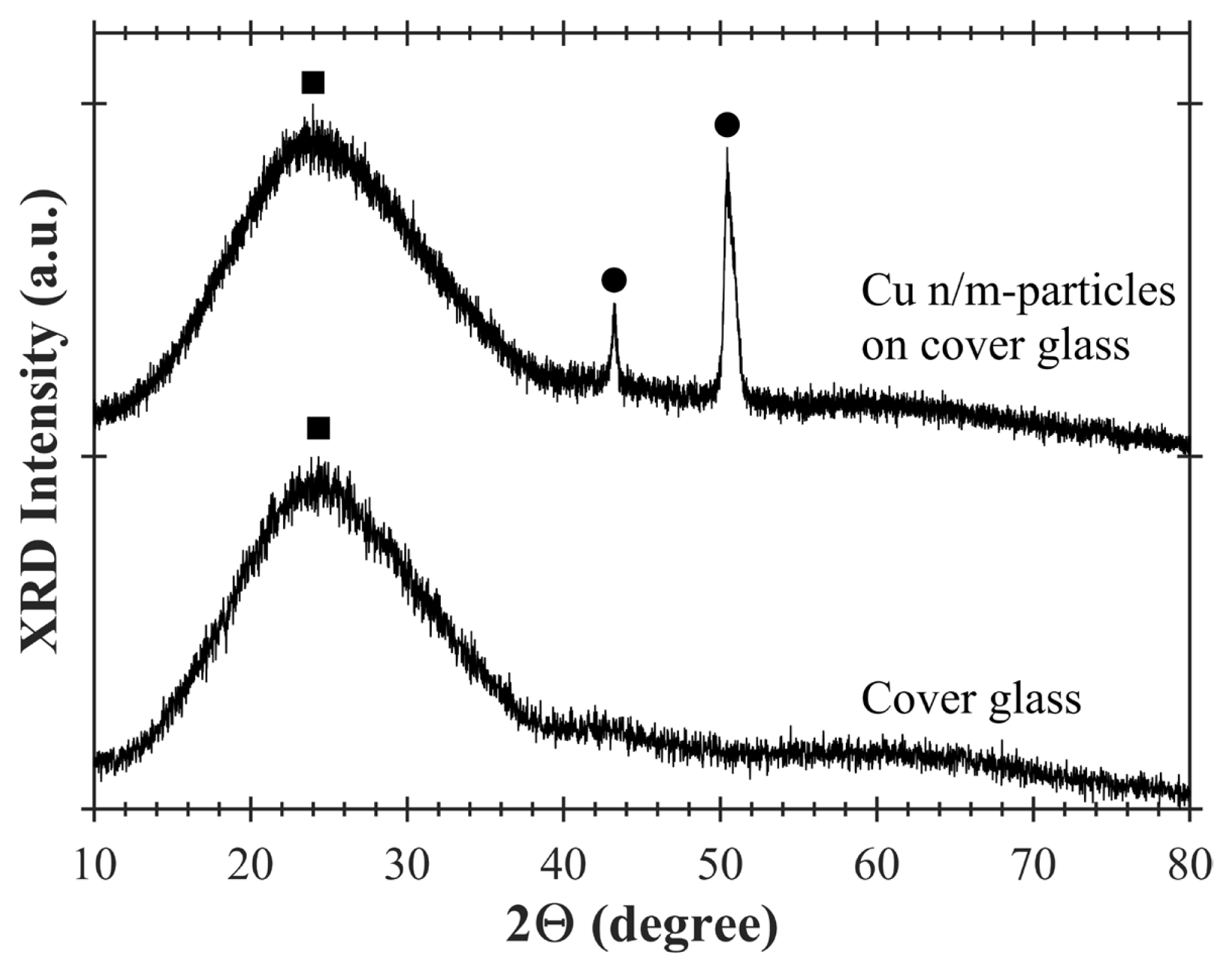

Supplement: Figure S2 — XRD spectra of cover glass and copper nano/microparticles (Cu n/m-particles) on cover glass. Black squares (■) indicate amorphous SiO2 XRD peak of cover glass. Black dots (●) show pure copper XRD peaks of nano/microparticles. [file turkjchem-47-3-616s2.tif]

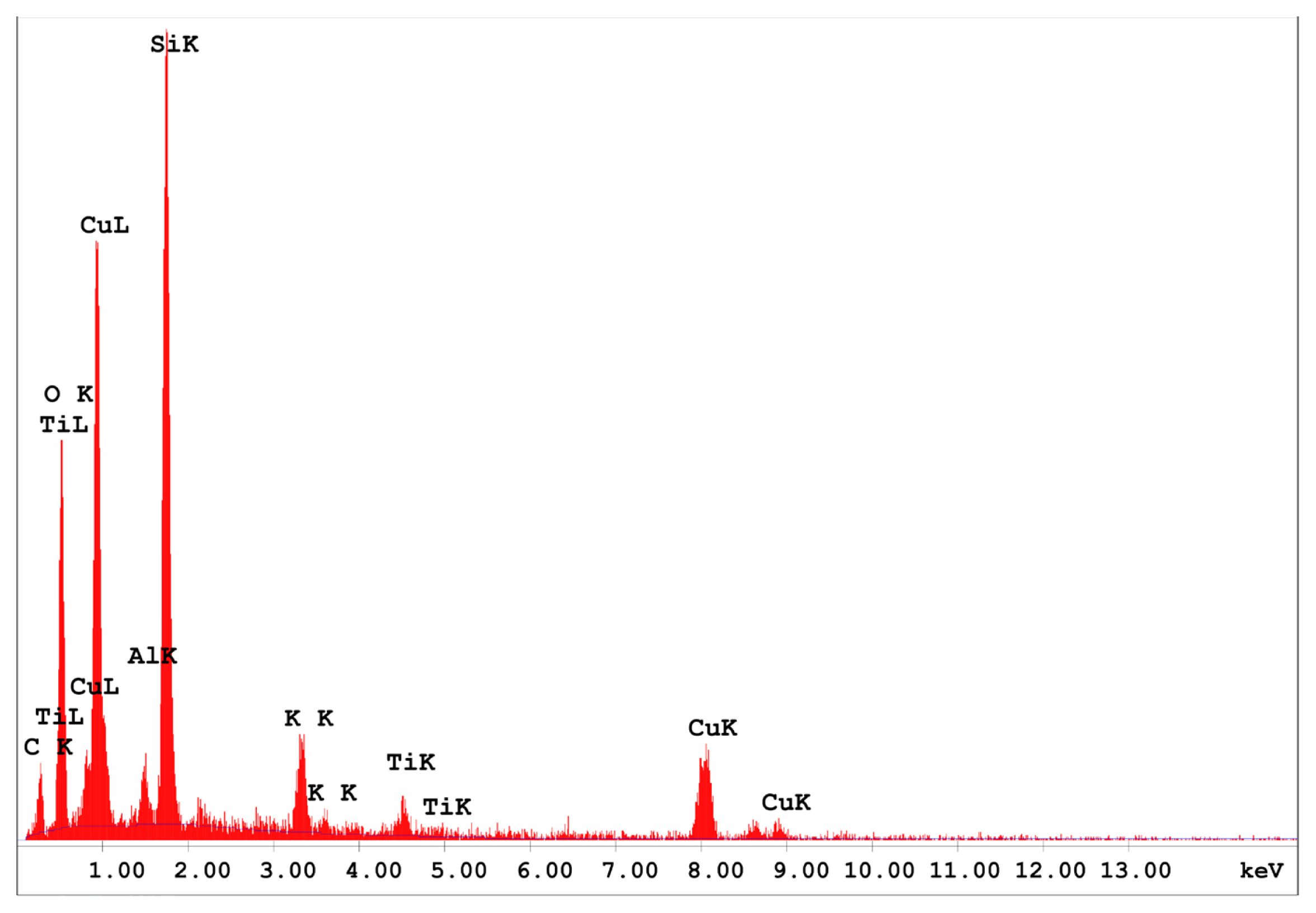

Supplement: Figure S3 — SEM-EDX spectrum of copper nano/microparticles on cover glass. Cu peaks are seen. [file turkjchem-47-3-616s3.tif]

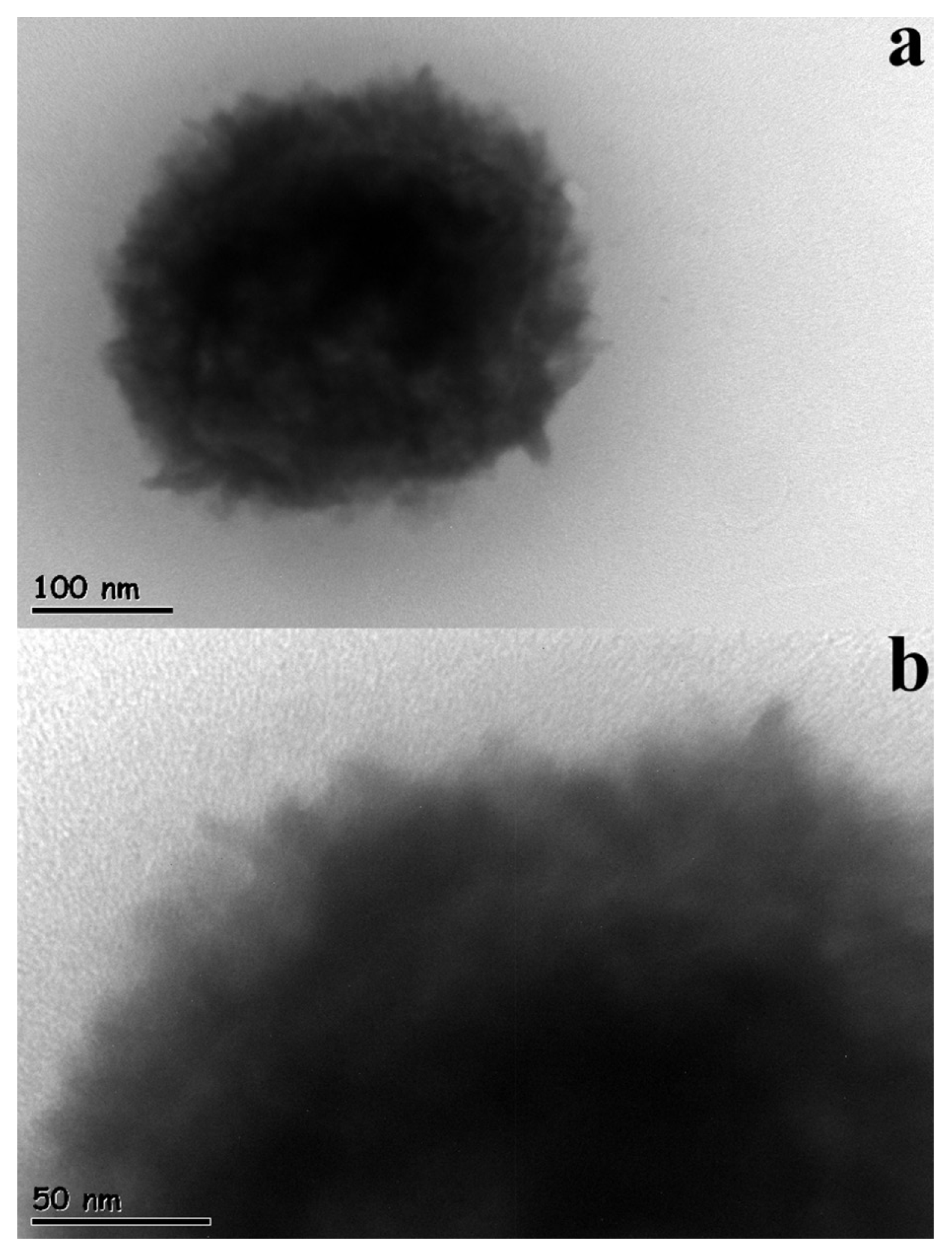

Supplement: Figure S4 — TEM photos of a copper nano/microparticle. a) Scale bar is 100 nm, and b) Scale bar is 50 nm. This copper nano/microparticle was precipitated from the reaction solution by centrifugation process. [file turkjchem-47-3-616s4.tif]

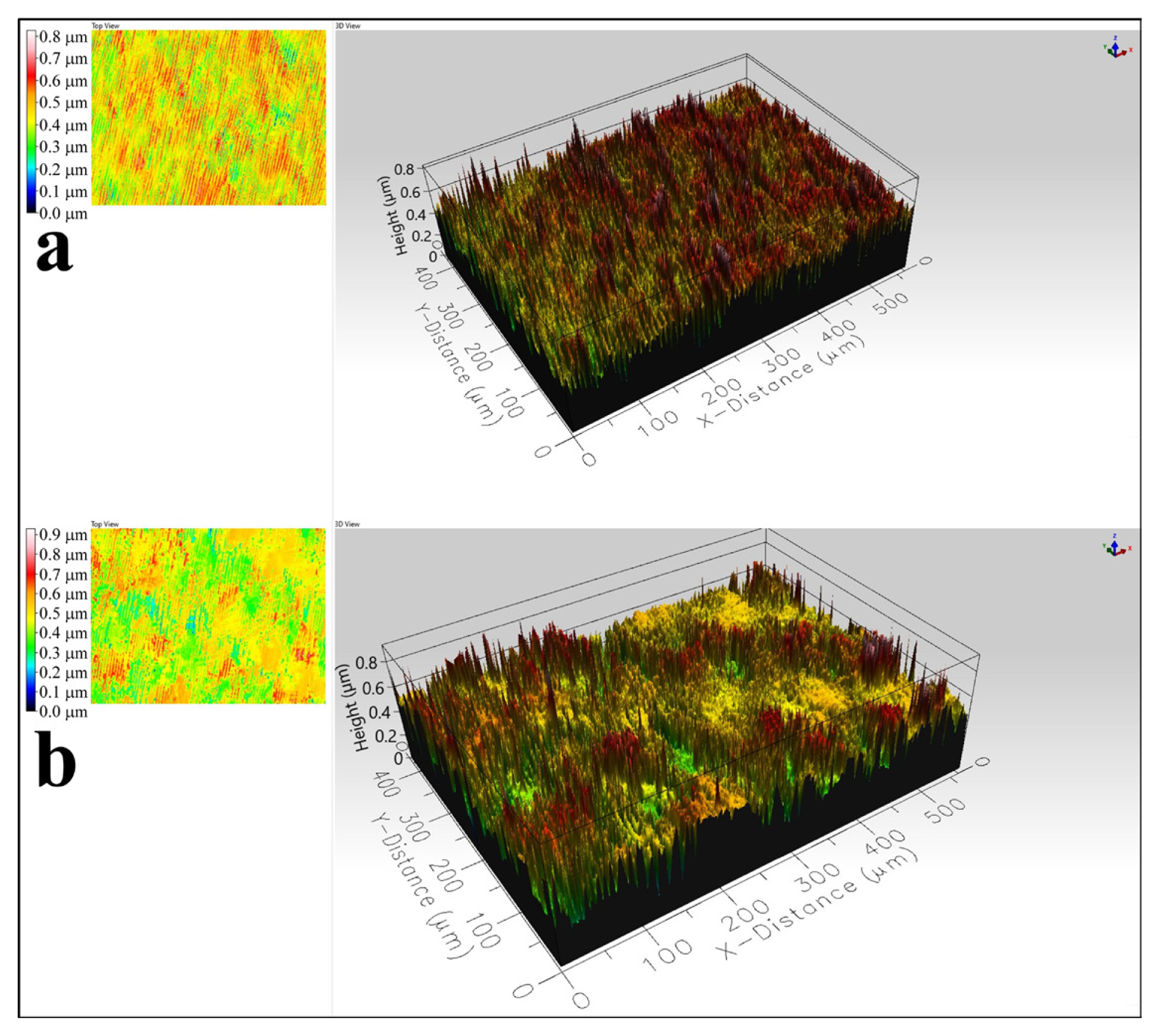

Supplement: Figure S5 — Film thicknesses of copper and copper oxide nano/microparticles on cover glass. a) Film thickness of Cu n/m-particles, and b) Film thickness of (86% Cu2O+14% CuO) n/m-particles. Vertical colour bars given at the left show film heights and each colour corresponds to a different height. Surface morphologies of these deposited films (coatings) were measured by using Filmetrics Profilm3D optical profilometer. A Gaussian-Lorentzian function is described at Equation (S1). Here, this equation represents a XRD peak in the context of this paper. [file turkjchem-47-3-616s5.tif]
